# Biosynthesis of Crystalline Silver and Gold Nanoparticles by Extremophilic Yeasts

**DOI:** 10.1155/2011/546074

**Published:** 2011-09-08

**Authors:** Ana Mourato, Mário Gadanho, Ana R. Lino, Rogério Tenreiro

**Affiliations:** ^1^Centro de Química e Bioquímica, Faculdade de Ciências, Universidade de Lisboa, Edificio C8, Campus da FCUL, Campo Grande, 1749-016 Lisboa, Portugal; ^2^Centro de Biodiversidade, Genómica Integrativa e Funcional (BioFIG), Faculdade de Ciências, Universidade de Lisboa, Edificio ICAT, Campus da FCUL, Campo Grande, 1749-016 Lisboa, Portugal

## Abstract

The biosynthesis of Ag and Au nanoparticles (NPs) was investigated using an extremophilic yeast strain isolated from acid mine drainage in Portugal. Three distinct studies were performed, namely, the growth of yeast strain in presence of metal ions, the use of yeast biomass for the metal nanoparticles synthesis, and of the supernatant obtained after 24-hour incubation of yeast biomass in water. The extremophilic strain under study was able to grow up to an Ag ion concentration of 1.5 mM whereas an increase of Au ion concentration over 0.09 mM caused a strong inhibitory effect. A successful route for the metal NPs synthesis was obtained using the yeast biomass. When the washed yeast cells were in contact with Ag or Au solutions, AgNPs smaller than 20 nm were produced, as for the AuNPs diameter ranged from 30 to 100 nm, as determined through transmission electron microscopy and confirmed by energy-dispersive X-ray spectra. The supernatant-based strategy provided evidence that proteins were released to the medium by the yeasts, which could be responsible for the formation and stabilisation of the Ag NPs, although the involvement of the cell wall seems fundamental for AuNPs synthesis.

## 1. Introduction

Nowadays, research in nanotechnology deals with the development of eco-friendly processes for the synthesis of stable nanoparticles, possessing well-defined shapes, and controlled narrow sizes [1]. Additionally, due to a vast demand for precious metals in fields such as electronics and catalysis, recovery of silver and gold from both primary and secondary sources is of most significance. Microbial recovery of precious metals with the formation of their nanoparticles is a green alternative to the conventional methods, and therefore it fulfils both issues aforementioned [2, 3]

Many biological systems such as bacteria [2, 4, 5], fungi [6], yeast [7, 8], and plants have been used for the biosynthesis of gold and silver nanoparticles, with well-defined size and distinct topography. Sastry et al. [9] have found that the acidophilic fungus *Verticillium *sp*., *when treated with an aqueous solution of Ag^+^ ions, resulted in the *in situ* reduction and consequent intracellular formation of AgNPs with good monodispersity. Moreover, Sanghi and Verma [10] showed that Ag^+^ ions in solution get adsorbed on the mycelium surface of the fungus *Coriolus versicolor* and are reduced *in situ* to Ag (0). According to Gericke and Pinches [11], the yeast *Pichia jadinii* (formerly *Candida utilis*), a fungal isolate from a metal-rich dump (isolate 6–3), and the fungus *Verticillium luteoalbum* present the ability to produce gold nanoparticles. Various particle morphologies, which included spherical, triangular, and hexagonal among other shapes, were obtained, and the particle size varied from a few to approximately 100 nm in diameter. Similar results were obtained when cells of the yeast *Yarrowia lipolytica* were placed in contact with gold ions, at pH 2 [12]. Lin et al. [8] also described that Au ions were bound to the cell wall of dead cells of the yeast *Saccharomyces cerevisiae* and then *in situ* reduced.

In this paper, we explore for the first time the potential of a yeast strain, isolated from acid mine drainage in Portugal (São Domingos, Alentejo), to reduce silver and gold ions to NPs. In order to accomplish this goal, the biosynthesis was explored by three distinct experimental strategies: (a) during yeast growth in presence of metal ions; (b) using yeast biomass obtained after 4 days of incubation; (c) using the supernatant obtained after 24 h of incubation of yeast biomass in water. The biosynthesised nanoparticles were characterized by a systematic spectroscopic and microscopic study. The influence of Ag and Au ions on the yeast growth is also addressed, as well as the role of the reducing sugar glucose on the formation of nanoparticles during yeast growth.

## 2. Experimental

### 2.1. Organisms and Growth Conditions

The yeast strain used in the present study was isolated from an acid mine drainage in Portugal. Cultures were maintained on MYGP agar comprising (gL^−1^): malt extract (Fluka), 7.0; yeast extract (Difco), 0.5; bacteriological agar (Biokar diagnostics, E), 15.0; neutralized bacteriological soya peptone (Fluka) 2.5. 

For experimental purposes, cultures were grown in YNBG liquid medium comprising (gL^−1^): yeast nitrogen base (Sigma), 67.0; D-glucose, (Merck), 20.0 (adapted from [12]). The pH was adjusted to 2.5. All incubations were performed at 22°C on an orbital shaker (160 rpm).

To examine the influence of silver or gold ions on yeast growth, cells suspensions (O.D. at 610 nm = 0.1) were prepared from 48 h starter cultures in modified YNBG (5% glucose). The culture suspensions were inoculated (1 *μ*L loop) into microplates with 300 *μ*L/well of YNBG growth medium containing silver (up to 3.0 mM; AgNO_3_, Merck) or gold (up to 1.5 mM; HAuCl_4_, Aldrich) ions. The yeast strain was cultivated in the automated Bioscreen C system (Lab systems Helsinki, Finland) at a controlled temperature of 22°C. The optical density of the cell suspensions was measured automatically at 610 nm in regular intervals of 2 h, for six days. Before each measurement, the culture wells were automatically shaken for 1 min. The experiments were carried out in duplicate. 

For the synthesis of nanoparticles using yeast biomass and yeast supernatant, the strains were grown in YNBG liquid medium at 22°C under shaking at 160 rpm for 96 h. The yeast biomass was separated from the culture broth by centrifugation (5000 rpm, at 10°C for 10 min) and washed thrice with sterile distilled water. Portions of the wet biomass (0.15 g) were then exposed to the metal ion solutions (1 mM AgNO_3_ or 0.6 mM HAuCl_4_) or resuspended in sterile distilled water and placed in a shaker at 22°C (160 rpm) for 24 h. The aqueous suspensions were then centrifuged (5000 rpm, at 10°C for 10 min) and aliquots of the supernatant were also placed in contact with the metal ions solutions (1 mM AgNO_3_ or 0.6 mM HAuCl_4_) for 24 h.

### 2.2. Instruments and Characterization

The metal ion reduction was monitored by measuring the UV-Vis spectra of the solution by periodic sampling of 2 mL aliquots of the aqueous component. The UV-Vis spectroscopy measurements were recorded on a Jasco dual-beam spectrophotometer (model V-560) operated at a resolution of 2 nm. For the XRD measurements, films of nanoparticles were made by drop-coating the metal nanoparticle/biomass suspension on amorphous Si substrates. X-ray diffraction (XRD) patterns were recorded in the transmission mode on a Philips PW 1830 instrument operating at 40 kV and a current of 30 mA with Cu *K*_*α*_ radiation (*λ* = 1.5404 Å).

For the transmission electron microscopy (TEM) analysis, the samples were immobilized on formvar-coated copper grids (200 *μ*m × 200 *μ*m mesh size) and the instrument used was a Hitachi H-8100 electron microscope operated at an accelerated voltage of 200 kV, coupled with an energy dispersive X-ray spectrophotometer. For the determination of average edge of lengths of metal nanoparticles in a given sample, approximately 110 nanoparticles per sample were taken into account.

FTIR measurements were carried out on a Satellite FTIR Matson spectrophotometer operating in transmittance mode at a resolution of 4 cm^−1^. A drop of the metal nanoparticle/supernatant suspension was placed between two calcium fluoride windows which were held in a Omni-cell (Specac).

The open circuit potential, OCP, was monitored during the biosynthesis of Au and Ag nanoparticles (24 h) using a solution containing washed biomass and metal ions, under constant stirring. A Pt foil (Area = 2 cm^2^) and a saturated calomel electrode (SCE) were used as working and reference electrode, respectively, in a one-compartment glass cell. A CHI Electrochemical Analyzer—620A Model controlled by a computer was used in the electrochemical experiments.

## 3. Results and Discussion

### 3.1. Influence of Metal Ions on the Growth of an Extremophilic Yeast Strain

For this study, the strain was grown in presence of both metal ions, Ag^+^ and Au^3+^, at different metal concentrations. All experiments included a negative control (well containing metal ions and nutrient medium, devoid of inoculum) and a positive one (well containing inoculum and nutrient medium, devoid of metal ion). Growth was observed in all positive controls confirming the viability of the yeast inocula. 

For yeast growth analysis, the area under each curve (AUC) was calculated by integration of the optical density with time in a 6-day period, since AUC is described as sensitive to the differential effects of the lag phase, the rate of growth and the maximum absorbance obtained during the incubation time [13]. For each experimental condition, the net area under curve (NAUC) was then obtained by subtracting the AUC of the negative control from the respective AUC. 

**Figure 1:**
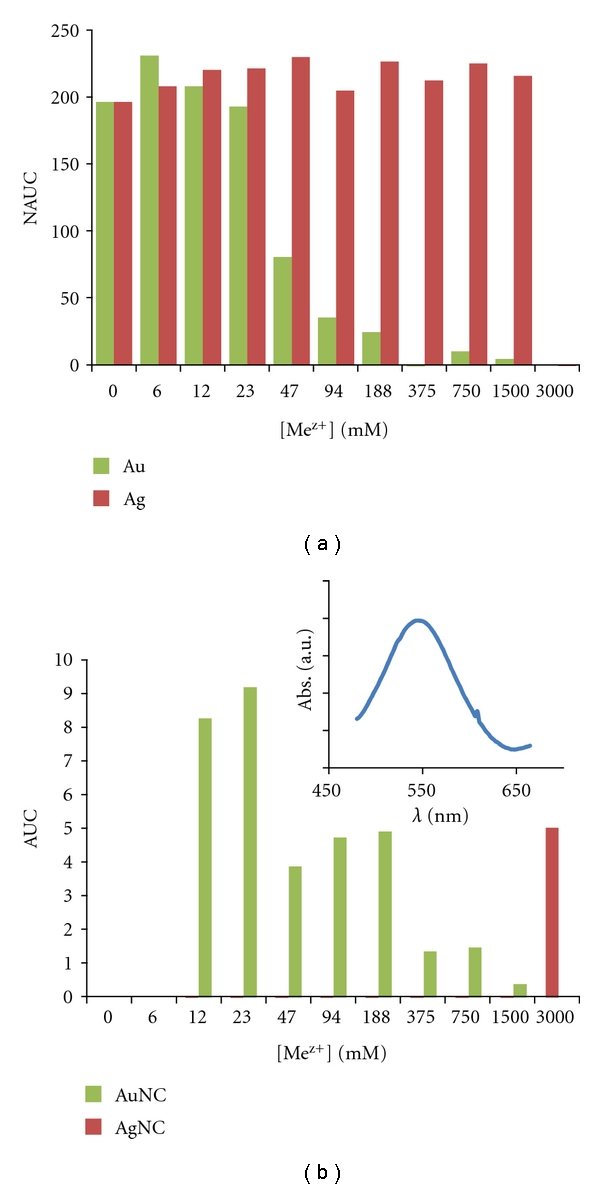
Influence of silver and gold concentration on (a) yeast growth and (b) in uninoculated growth medium (glucose). Measurements were performed at OD 610 nm and NAUC (net area under curve) values refer to 6 days. Inset: UV-Vis spectra recorded after the reaction of uninoculated growth medium with 1 mM HAuCl_4_ for 24 h.

The NAUC values determined in presence of different concentrations of metal ions are depicted in Figure 1. In presence of silver ions, the yeast growth occurred even when Ag concentration was as high as 1.5 mM whereas growth was not observed for 3.0 mM (Figure 1(a)). In a recent study [14], the potential toxicity of commercial AgNPs and ions on the growth of yeasts (*S. cerevisiae*), gram-negative bacteria (*Escherichia coli*), and gram-positive bacteria (*Bacillus subtilis*) was investigated, were a higher survival rate for the yeast was observed as compared with bacteria. 

Regarding Au ions, a strong inhibitory effect was observed with the increase of concentration, which is more pronounced for concentrations higher than 90 *μ*M Au (Figure 1(a)), pointing to the higher toxicity of this metal when compared with silver. 

The AUC values obtained for the negative control, at different concentrations of metal species, are presented in Figure 1(b). Although interaction with Ag ions is only noticed for the highest concentration (3.0 mM), the results show that Au ions react with the growth medium, most probably with glucose, which can be oxidised towards gluconic acid promoting the reduction of gold ions to the elemental form, with concomitant NPs formation. In fact, it is reported that glucose can be an effective reducer of trivalent gold [15, 16].The inset of Figure 1(b) shows the UV-Vis spectra obtained after the reaction of uninoculated growth medium (glucose) with 1 mM HAuCl_4_ for 24 h, where an absorption band centered at 545 nm is shown, suggesting the formation of spherical AuNPs in solution. Consequently, the glucose availability as nutrient diminishes and a possible consequence is the decrease on the yeast growth rate. 

### 3.2. Synthesis of Silver and Gold Nanoparticles by Yeast Biomass

**Figure 2:**
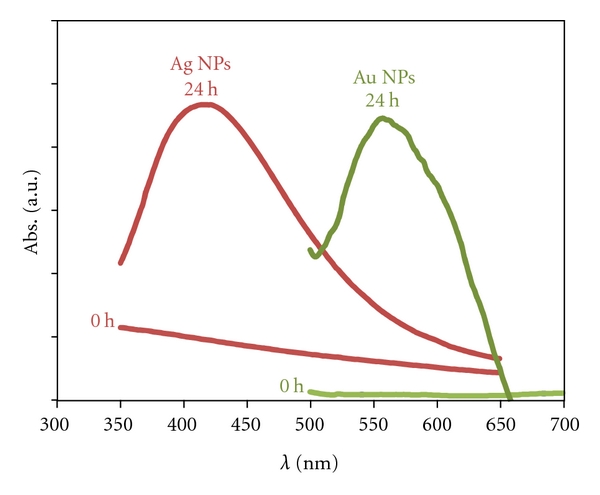
UV-Vis spectra recorded with respect to time after the reaction 0 h and 24 h of contact with yeast biomass with 1 mM AgNO_3 _(red line) and 0.6 mM HAuCl_4_ (green line).

The aim of the study presented in this section was to evaluate if the yeast biomass (after removal from the culture medium and washing with water) was able to produce metal nanoparticles when in contact with metal ions. The UV-Vis spectra recorded after 24 h of reaction between yeast biomass and Ag/Au ions are plotted in Figure 2. The spectra show well-defined surface plasmon resonance bands centered at *ca*. 420 nm and 550 nm, characteristic of AgNPs and AuNPs, respectively.

**Figure 3:**
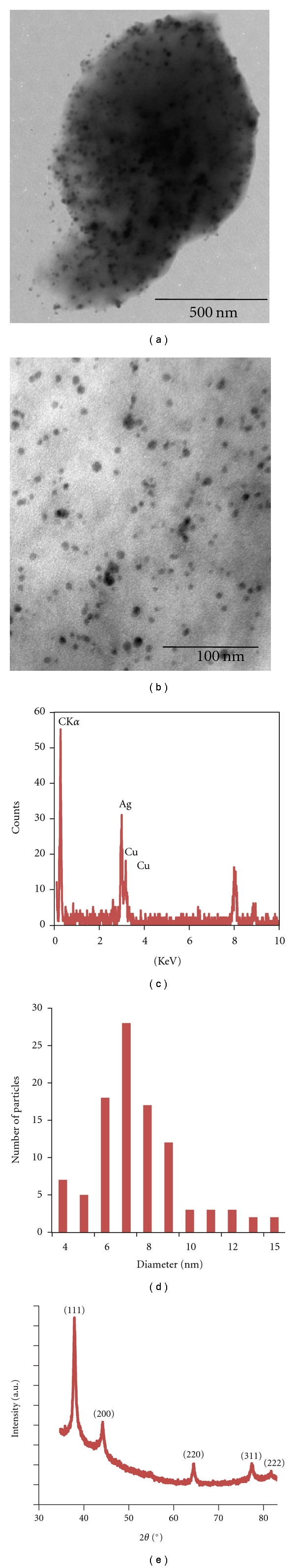
TEM micrographs recorded on a drop-coated film with yeast biomass/AgNPs aqueous suspension, after 24 h (a). EDX spectra (b) and particle size distribution histogram determined from the TEM micrographs (c). XRD pattern recorded from the thin film prepared by drop coating the biomass/AgNPs aqueous suspension after 24 h on a Si wafer; the principal Bragg reflections are identified (d).

Representative TEM images recorded from the AgNPs/yeast film deposited on a formvar-coated copper TEM grid are shown in Figures 3(a) and 3(b). These images show individual spherical Ag particles, distributed in/on the yeasts, and the formation of extracellular nanoparticles. Even so, the possibility of intracellular synthesis of the nanoparticles cannot be disregarded. The EDX spectrum recorded during the TEM analysis of these assemblies (Figure 3(c)) shows the presence of silver as well as carbon and copper from TEM grids. It is worth to mention that the AgNPs suspension is stable even after 1 month. The histogram of the AgNPs size distribution, obtained by the analysis of the TEM images, is represented in Figure 3(d). The particle core (Ag) diameters were found to be in the range of 4 to 15 nm, and the average core diameter was estimated to be 7.5 nm (obtained by analyzing ca. 110 particles using standard image analysis software). In Figure 3(e), the XRD diffractogram obtained for the biomass/AgNPs suspension is depicted. All the direction lines were indexed according to JCPDS-ICDD data [17]. The characteristic peaks for polycrystalline silver appeared at 38.1°, 44.3°, 64.4°, 77.4°, 81.5°, 98.7°, 110.9°, and 114.9°, corresponding to crystal facets of (111), (200), (220), (311), and (222), respectively [17]. Each crystallographic facet contains energetically distinct sites based on atom density. 

**Figure 4:**
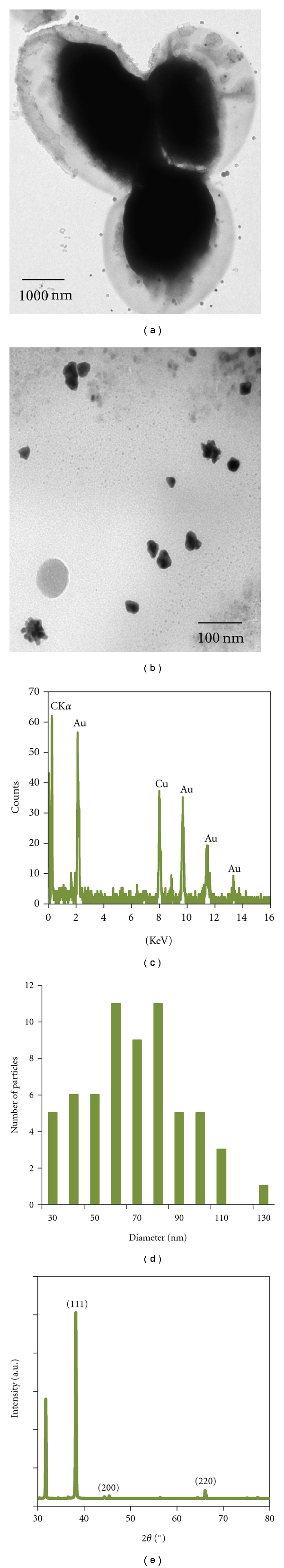
TEM micrographs recorded on a drop-coated film with yeast biomass/AuNPs aqueous suspension, after 24 h (a and b). EDX spectra (c) and particle size distribution histogram determined from the TEM micrographs (d). XRD pattern recorded from the thin film prepared by drop coating the biomass/AuNPs aqueous suspension after 24 h on a Si wafer; the principal Bragg reflections are identified (e).

The AuNPs biosynthesis was confirmed by TEM-EDX analysis as shown in Figures 4(a)–4(c). In the left picture (a) well-separated AuNPs with spherical morphology are evident nearby the yeast cell and on/in the cell. The right picture (b) shows aggregates of AuNPs with irregular shapes. Even though there is large-scale association of the particles, individual and discrete AuNPs can be discerned in this micrograph, and most probably, they are stabilized with proteins that prevent their sintering [12, 18, 19].

The EDX spectra (Figure 4(c)) confirmed the presence of Au (0) and the histogram, derived from the TEM images analysis, shows AuNPs with diameters ranging from 30 to 100 nm (Figure 4(d)). Further evidence of formation of elemental gold is provided by X-ray diffraction analysis of yeast biomass/AuNPs films prepared by drop coating on Si substrate (Figure 4(e)). The peaks due to (111), (200), and (220) Bragg reflection at 2*θ* = 38°, 45°, and 67°, respectively, were the only features observed corresponding to polycrystalline gold with face-centered cubic unit cell.

### 3.3. Role of Yeast Supernatant on Ag and Au Reduction

**Figure 5:**
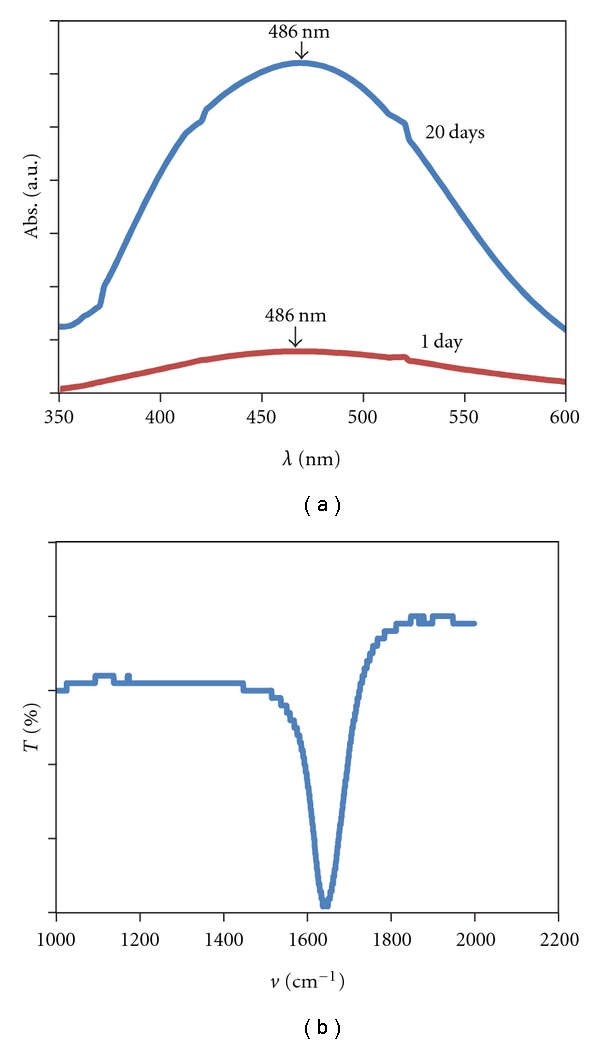
UV-Vis spectra recorded with respect to time after the reaction for 24 h and 20 days of contact of yeast supernatant with 1 mM AgNO_3_ (a). FTIR spectrums recorded from a drop-coated film of the AgNPs/supernatant (b).

In order to verify if the yeast under starving conditions release reducing agents into solution, which could be responsible for the formation of metal NPs, the supernatant, obtained after 24 h of yeast biomass incubation in water, was placed in contact with the metal ion solution. Indeed, AgNPs were formed by this experimental approach as shown by the presence of the plasmon resonance band in the UV-Vis spectra (Figure 5). The AgNPs suspension was stable even after 20 days, and the intensity increased, suggesting an augmentation in the number of nanoparticles in solution. The FTIR spectrum of the AgNPs/supernatant (Figure 5(b)) reveals the presence of one band at 1650 cm^−1^, assigned to carbonyl stretch in the amide linkages of the proteins [19]. From the results obtained it is possible to advocate that proteins are released by the yeasts, since they are present in the supernatant, and therefore responsible for the formation and stabilisation of the AgNPs. 

In the case of the supernatant in contact with the gold ions, the formation of AuNPs would be expected, since gold ions are reduced in mild conditions as compared with silver ions, according with the reduction potentials (1)–(3):

(1) $$\begin{array}{cc} & {\text{Ag}}^{+}+{\text{e}}^{-}\to \text{Ag}\\ & \;{\text{E}}^{0}=0.779\;\text{V}\;\text{versus}\;\text{NHE}, \end{array}$$

(2) $$\begin{array}{cc} & {\text{Au}{\text{Cl}}_{4}^{}}^{-}+\;{2\text{e}}^{-}\to {\text{Au}{\text{Cl}}_{2}^{}}^{-}+2{\text{Cl}}^{-}\\ & \;{\text{E}}^{0}=0.926\;\text{V}\;\text{versus}\;\text{NHE}, \end{array}$$

(3) $$\begin{array}{cc} & {\text{Au}{\text{Cl}}_{2}^{}}^{-}+\;{\text{e}}^{-}\to \text{Au}\;+\;2{\text{Cl}}^{-}\\ & \;{\text{E}}^{0}=1.15\;\text{V}\;\text{versus}\;\text{NHE}. \end{array}$$

However, no evidence for the reduction of Au ions towards the formation of nanoparticles was observed. In this case, it can be hypothesized that the supernatant is constituted by compounds which had coordinated with the Au ions, and the complex formed has an reduction potential much more higher (more negative) than the silver and gold ions (Au^3+^, AuCl_4_^−^, AuCl_2_^−^). These results make a strong case for the role of the cell wall on the formation of AuNPs, since it only occurred when the metal ion solution was in contact with yeast cells. 

**Figure 6:**
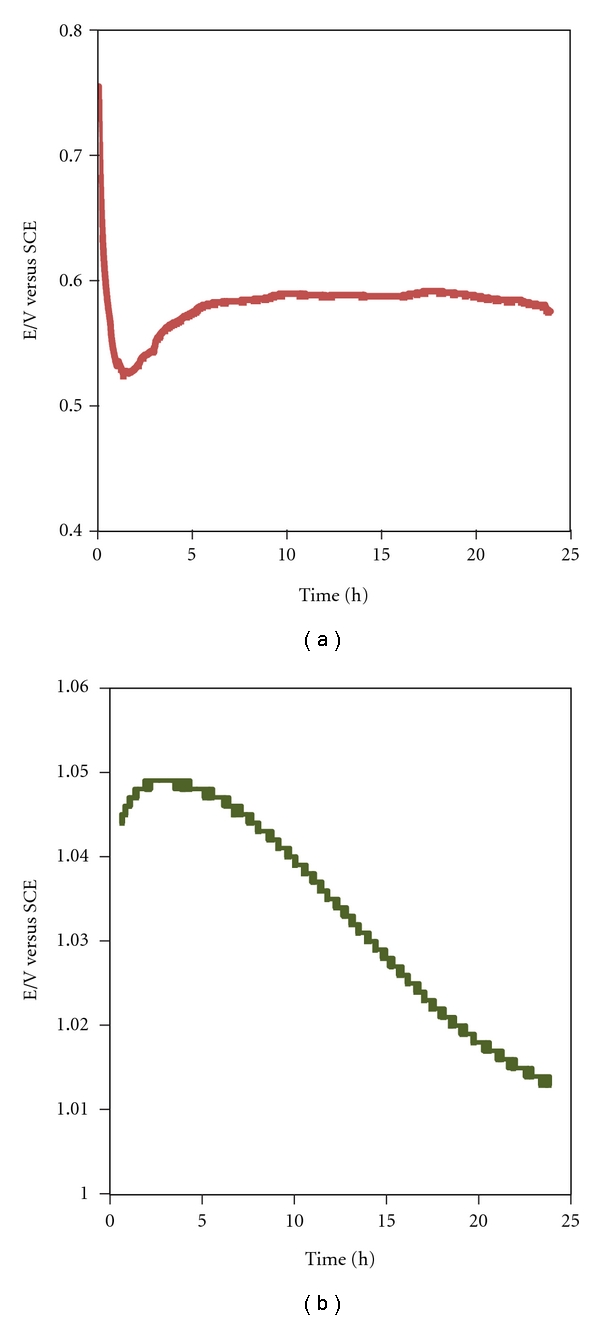
Open-circuit potential evaluation of the biomass suspension in presence of (a) Ag ions and (b) Au ions.

To get more insight on the reaction progress of Ag and Au reduction, an approach based on open-circuit potential measurements was followed (Figure 6). The value of the OCP depends on the reductant/ion metal ratio, C_R_/C_O_ where R-reductant and O-oxidant. In the present work C_R_ stands for reducing active sites on the yeast membrane and/or compounds released from the cells under stress conditions whereas the C_O_ corresponds to the metal ions. 

In the literature it is suggested that the reduction/formation of the metal nanoparticles involves trapping of the metallic ions on the surface of the cells via electrostatic interaction between them and negatively charged groups (e.g., carboxylate, hydroxyl) in enzymes or in negatively charged amino acids of polypeptides [20] present in the cell wall. Sastry et al. [9] proposed that, after electrostatic trapping, the silver ions are reduced by enzymes present in the cell wall leading to the formation of silver nuclei, which subsequently grow by further reduction of Ag^+^ ions. Also, it was postulated that silver ions could diffuse through the cell wall and were reduced by enzymes present on the cytoplasmic membrane and within the cytoplasm. This enzyme-based pathway on silver and gold nanoparticle synthesis has also been referred for distinct microorganisms by other authors [10–12]. In a recent study it was found that the assumed higher organic content leached out from inactive cells can be responsible for the higher and rapid productivity of Ag and Au NPs [21].

The initial potential value for AgNPs/supernatant was 0.755 V which is smaller than the potential required for the reduction of Ag ions (Figure 6(a)). Observed in the initial moments were an OCP decrease and the development of a minimum at 0.524 V which should be mostly related to the sudden and quick release of the reductive compounds into the solution (this process occurs within *≈* 1 h.30 m). In a second stage after the OCP minimum, the species released by the microorganism are oxidized with the simultaneous reduction of Ag ions producing silver nuclei/nanoparticles, and therefore the potential increases due to a still excess of metal ions in solution. In this stage, the silver ions would be consumed mainly on the formation of nuclei whereas the secondary reduction process which takes place on the surface of the preformed nuclei would be hindered. Afterwards, a plateau is reached, at OCP of 0.588, due to a relatively stable C_R_/C_O_ ratio, indicating that the relative amount of active sites on the membrane/reducing compounds and silver ion remains constant, simultaneously, also in this stage Ag nuclei growth can take place.

In the suspension containing Au ions, the initial OCP increased from 1.032 to a maximum of 1.131 V, after almost 3 hours. Subsequently, a gradual potential decrease until a plateau at 1.011 V is developed is observed. The OCP profile depicted for the Au ion bioreduction and the small variation of potential supports that only the membrane is involved on the process, since the small increase of potential towards the maximum value can be attributed to the Au ions species present in excess as compared with reducing agents. The slow decrease of potential with time reflects the slow adsorption to the binding sites of the membrane and reduction of the Au ions leading to the nuclei formation. The plateau can be related with the limited number of active sites available and/or the growth of the Au nuclei to particles with average diameters of ca. 15 nm. Additionally, the possibility that Au ions could form a stable complex with compounds released from the microorganisms cannot be disregarded. Indeed, it can explain the small potential difference observed in the initial moments as compared with the end of reaction.

## 4. Conclusions

It has been demonstrated that extremophilic yeasts, isolated from acid mine drainage in Portugal, are able to grow in presence of Ag and Au ions and can be exploited to produce nanocrystalline silver and gold particles. By using microorganisms for NP synthesis, advantage of a natural detoxification bioprocess can be withdraw which can be considered as an eco-friendly contrary to the classic AuNP synthesis methods that require a myriad of expensive and toxic reducing, stabilizing, and functionalizing chemicals.

The resulting Ag and Au nanoparticles displayed controllable structural and optical properties as demonstrated by UV-vis spectroscopic studies, TEM observations, and XRD data. The results indicate that the Ag and Au nanoparticles formation could take place intracellularly and/or extracellularly. The OCP data suggests that the cell wall plays an important role for the biosynthesis of both metal nanoparticles whereas the compounds released by the yeasts were only able to reduce the silver ions. AgNPs displayed an average diameter of 20 nm whereas the size range of the AuNPs was ca. 20 to 100 nm. The nanoparticles were well dispersed, indicating that they are capped by stabilizing agents, most probably by proteins.
